# Effects of Environmental Enrichment on Dog Behaviour: Pilot Study

**DOI:** 10.3390/ani12020141

**Published:** 2022-01-07

**Authors:** Rebecca L. Hunt, Helen Whiteside, Susanne Prankel

**Affiliations:** 1 Guide Dogs National Centre, Banbury Road, Bishop’s Tachbrook, Warwickshire CV33 9WF, UK; helen.vaterlawswhiteside@guidedogs.org.uk; 2 School of Science and the Environment, University of Worcester, St John’s. Campus, Henwick Grove, St. John’s, Worcester WR2 6AJ, UK; s.prankel@worc.ac.uk

**Keywords:** environmental enrichment, dog, behaviour

## Abstract

**Simple Summary:**

The quality of life for domestic and captive animals can be enhanced and improved by providing additional stimuli and activities, known as environmental enrichment (EE). However, it is important to ensure the enrichment provides benefit to the animal, and as such the behaviour of the animals can be monitored to understand the activities’ impacts. A pilot study was undertaken to evaluate the impact of seven different EE activities on the behaviour of 10 training dogs housed in an office environment during training within an assistance dog charity. Results suggest there is a need to diversify thinking around EE, shifting common thinking of EE as one entity to instead consider EE in different categories and to ensure animals receive a mixture of EE types.

**Abstract:**

Environmental enrichment (EE) can be used to enhance the environment of various animals. The aim of this pilot study was to determine the effects of seven EE activities (Bonding, Bubble machine, Conspecific play, Interactive toy, Playhouse, Stuffed food toy and Tug play) on dog behaviour, pre- and post-EE for dogs housed in an office environment during training as part of an assistance dog training programme. EE activities resulted in a significant increase in the frequency of relaxation behaviours (*p* < 0.01) and a significant reduction in alert (*p* < 0.01) and stress behaviours (*p* = 0.02). Results suggest various benefits of the different activities with Conspecific Play and Playhouse activities having the greatest overall positive behaviour change when compared to the other activities. The food-based EE activities (Interactive toy and Stuffed food toy) had the least behaviour change of all the activities provided. Findings will be of interest to pet owners, animal rescue centres, dog trainers and working dog organisations.

## 1. Introduction

There are many definitions of environmental enrichment (EE) within the literature, but EE is commonly described as a technique designed to enhance the quality of life in captive and domestic animals by providing additional and temporary environmental stimuli to promote psychological and physiological wellbeing [1,2,3]. EE has been shown to be beneficial to the wellbeing of species including rats [4], pigs [5], cats [6] and geckos [7].

EE is used as a standard part of dog husbandry across a range of environments including the home [8], kennels [9,10,11,12,13] and laboratories [14]. The reported benefits of EE for dogs include reduced stress [10,15], decreases in stereotypic and abnormal behaviours [10,12,14], increased relaxation [16], improved cognitive abilities [3,13] and reduced barking or vocalisations [13,15,16]. Recently there has also been an increase in the number of studies reporting or reviewing the use of different EE types (e.g., the use of olfactory [16,17] and auditory EE [16,18]) to improve dog welfare, particularly in rehoming or shelter environments [19].

To support the development of best practice guidance for clinicians, practitioners and pet dog owners, a pilot study was undertaken to evaluate the impact of seven different EE activities on the behaviour of 10 training dogs in an office environment within an assistance dog charity. The aim of this study was to examine the behaviour of dogs pre- and post-enrichment.

## 2. Materials and Methods

A group of assistance dogs in training were the subjects of this study. A total of 10 neutered dogs (4M/6F) aged 12 to 14 months from two breeds (nine Golden Retriever _Sire_ × Labradors _Dam_, and one German Shepherd Dog _Sire_ × Golden Retriever _Dam_) were bred and reared as part of an assistance dog training programme. Prior to entering training, the dogs were raised by volunteer puppy raisers from eight weeks of age. Throughout training, the dogs were housed in an office environment when not undertaking formal training, and during the evenings and weekends were housed in volunteer homes.

Data were collected over a period of eight weeks. Individual dogs were exposed to each of the seven EE activities (Table 1) twice during the study in a randomised order. Activities were performed in an allocated activity room (approximately 5 m × 4 m in size) familiar to the dogs, with the exception of the stuffed food toy which was given to subjects in their pen. Dogs were supervised at all times during the activities by the assessor. Behavioural data were collected using continuous video recordings of the dogs in their individual pen for two 15-min time periods; one prior to the activity (pre-EE) and another post-activity (post-EE). The time of 15 minutes was chosen to align with the time (approximately one-hour slots) that dogs would spend in a pen when not undertaking training.

Behavioural frequencies were recorded using an ethogram. Behaviours that were displayed for up to 15 s were included as one count. For any behaviour that was continuously displayed beyond 15 s additional counts were recorded. Related behaviours were grouped under six behavioural categories (maintenance, play, locomotion, relaxation, alert and stress; see Supplementary Table S1 for behaviours). The same observer (the corresponding author) was used for the creation of the ethogram and all behavioural analyses to exclude assessor bias. Frequencies of behaviours were averaged from the two observations to create a single pre and post EE value for each category of behaviour per dog. An overall behavioural change score for each behaviour category was then calculated by subtracting the pre-EE value from the post-EE value for each dog. As such, a negative behavioural change score was associated with a decrease in the frequency of the behaviour post-EE activities. A positive behavioural change score was associated with an increase in the frequency of the behaviour post-EE activities.

To establish the impact of EE type on each behavioural category, general linear mixed effect models were run with EE type as a fixed effect and dog as a random effect. LSD post hoc pairwise analyses were used to compare the behavioural change scores between each EE type following a significant result. Frequencies were reported as mean ± one standard error. Results were considered significant when *p* < 0.05. All analyses were carried out using IBM SPSS Statistics for Windows version 22.0 (IBM Corp, Armonk, NY, USA).

The research project was approved by The University of Worcester’s Ethics Committee. All procedures adhered to the assistance dog organisation’s welfare and ethical policies. Dog behaviour was continuously monitored throughout all EE activities with a plan for any activity to be stopped should a dog show any signs of distress.

## 3. Results

**Figure 1:**
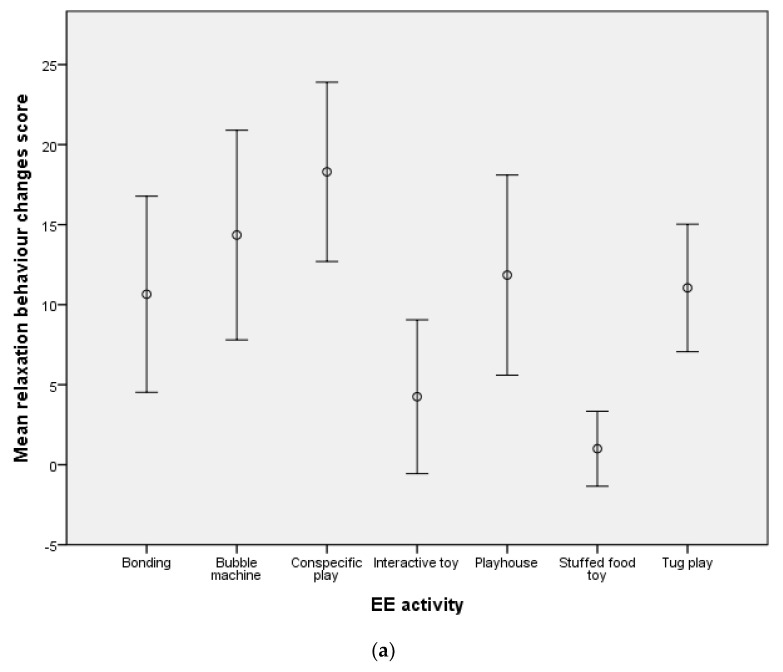

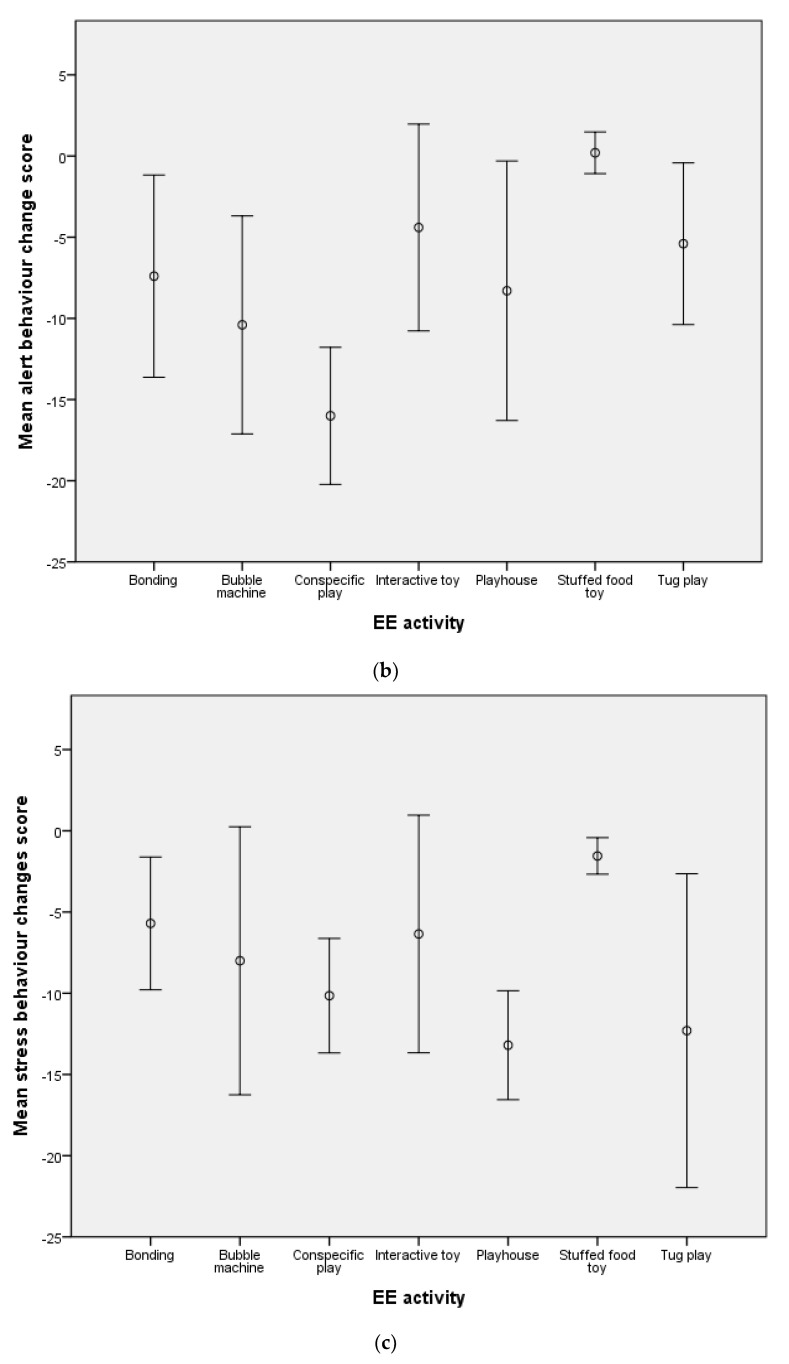
Mean behavioural change scores for (**a**) relaxation (**b**) alert and (**c**) stress behaviours for the seven EE activities. 95% CI bars are shown.

Due to the infrequent display of maintenance (with the exception of drinking post-EE), play and locomotion behaviours during pre- and post-EE videos (see Supplementary Table S2), the behaviours were not included in further statistical analyses. EE activities resulted in a significant increase in the frequency of relaxation behaviours (d.f. = 6, F = 6.48, *p* < 0.01; Figure 1a) and a significant reduction in alert (d.f. = 6, F = 4.16, *p* < 0.01; Figure 1b) and stress behaviours (d.f. = 6, F = 2.79, *p* = 0.02; Figure 1c). For mean behavioural change scores for each EE activity and behaviour category, see Supplementary Table S2.

A visual representation of the significant behaviour changes for each EE activity when compared to the other activities is shown in Table 2. Relaxation behavioural change scores were significantly higher for all activities when compared to the food-based EE activities (Stuffed food toy and Interactive toy). Alert behavioural change scores were significantly lower for Conspecific play when compared to all other EE except for the Bubble machine. There was also a significant reduction in alert behaviours for Bonding, Bubble machine, Conspecific play and Playhouse when compared to the Stuffed food toy. Stress behavioural change scores were significantly lower for Conspecific play, Playhouse and Tug play when compared to the Stuffed food toy. Additionally, the Playhouse activity resulted in significantly lower stress behavioural change scores compared to Bonding. There were no other significant differences between the EE types and behavioural change scores. For significance values for all comparisons, see Supplementary Table S3.

## 4. Discussion

For an environmental intervention to be considered enriching, the changes it produces must be linked to an improvement in an animals’ state. Therefore, measuring behaviours is essential when examining whether environmental interventions can be considered successful [20]. Relaxation, alert and stress behaviours in dogs are widely used indicators of psychological welfare [21,22,23]. Despite the importance of EE, there are few controlled studies assessing the effect of multiple enrichment activities on dog welfare in different environments [24]. This pilot study presents the first results examining the impact of individual EE activities on dog behaviour in an office environment within an assistance dog training programme. Findings from this study suggested that Conspecific play and Playhouse activities resulted in the greatest behaviour change compared with all other activities. The smallest behaviour change overall was observed for food-based activities (Stuffed food toy and Interactive toy).

The impact of EE activities on dog behaviour varies dependent upon the type of activity and the animal’s mental state. Food-based EE activities are frequently provided for dogs [10,13,25], yet the relative welfare benefits reported are varied. A recent study reported feeding enrichment to result in reduced stereotypies and increased activity levels for kennelled dogs [26]. Similarly, Schipper et al. [13] found stuffed food toys and treat games reduced barking frequencies and increased activity levels for dogs housed in kennels. However, for some working dogs it would appear that the feeding enrichment is not as impactful. Gaines et al. [25] suggested a stuffed food toy had minimal impact on the behaviour and working performance of military dogs and so was of limited welfare value. Similarly, this study found the food-based EE activities (Stuffed food toy and Interactive toy) to have the least behaviour impacts overall for a group of assistance dogs when compared to the other activities. The food-based EE items were removed from the dogs when all food had been eaten so the toys were not chewed. As such, the time spent interacting with the EE was variable for individual dogs depending on how quickly the food was eaten. The 15-min pre and post EE behaviour change remained consistent for all EE assessed. The dogs in this study were not permanently housed in kennels which may explain the reduced impact of food-based EE when compared to dogs housed permanently in kennels during training. The results for food-based EE may also vary by breed or individual dogs based on food motivation levels.

There are reported benefits for dogs when provided with social contact, either conspecifics or humans [27,28]. Social contact EE activities are associated with decreased stereotypy, greater sociability, reduced periods of inactivity, decreased cortisol concentrations and increased relaxation [29,30,31,32]. Wells and Hepper [27] found social stimulation had a greater positive impact on dog behaviour compared to the provision of toys, suggesting that providing a range of EE activities is likely to produce the greatest benefit. This study also supports these findings as the Conspecific play activity had the greatest overall behavioural changes for any EE activities (see Table 2 and Table S2). The variation in behavioural responses to different activities suggests that a combination of EE activities enables dogs to display a wider range of natural behaviours.

The Playhouse had the highest mean behavioural change score in stress behaviours and the Bubble machine had the second highest mean behavioural change scores for relaxation and alert behaviours (see Table S2) The Playhouse and Bubble machine were completely novel EEs provided to the dogs as part of the study. Providing new EE activities and rotating them is important to maximise benefits and prevent habituation [1]. It has also been suggested that if a break is provided from EE presentation, the effect of any habituation can recover, and the animal can successfully reinvestigate the EE [1]. The Bubble machine also added scent EE in the form of bacon scented bubbles. Studies have shown the benefits of utilising scent such as lavender [16,17] and rabbit scent [17]. EE activities that influence multiple senses could have additional positive impacts on behaviour.

There were some limitations to this study as it was a small pilot study including 10 dogs and therefore results should be interpreted with caution. As the study was a pilot, inter-rater reliability was not included due to data being collected by one assessor. It is recommended that future studies include inter-rater reliability measures. The method for collecting data on behaviour change scores could have been improved if the duration of behaviour display had been recorded. The authors suggest future studies on EE record the proportion of time spent displaying each behaviour and the time the dog spent engaging with each activity.

## 5. Conclusions

These results suggest there is a variation in the impact of EE type on behavioural displays. Therefore, by providing a range of different activities and rotating the activities in a random order, dogs are able to display a wider range of behaviours and potentially reduce the occurrence of habituation. However, more studies are required to compare the impact of specific EE activities on individual behaviours and thus determine their welfare value.

## Figures and Tables

**Table 1:** A description of the EE activities and handling protocol for assessment.

EE Activity	Handling Protocol
Bonding	Handler sits in room providing dog with physical contact, stroking and grooming for 15 min if dog chooses to engage in the interaction
Bubble machine	Bubble machine blowing bacon flavour bubbles placed in room with dog for 15 min
Conspecific play	Dog has supervised interaction for 15 min with a known conspecific
Interactive toy	Food puzzle game provided until all treats retrieved from toy or after 15 min
Playhouse	Playhouse consisting of tunnels, slides and platforms provided for the dogs to explore for 15 min with handler providing encouragement when needed
Stuffed food toy	Stuffed food toy provided until all food removed by subject or after 15 min
Tug play	Known handler engages in 15 min play with the dog through tug and fetch games using soft toys and rope knots

**Table 2:** Summary of behaviour change scores when comparing each EE activity to one another using LSD post hoc pairwise analyses for alert (A), relaxation (R) and stress (S) behaviour categories. A green square indicates a significant increase for relaxation behaviours or a significant decrease for alert and stress behaviours. A red square indicates a significant decrease for relaxation behaviours or a significant increase for alert and stress behaviours. A black square indicates the same activity, so no comparisons were undertaken.

EE Activity	Bonding			Bubble Machine			Conspecific play			Interactive toy			Playhouse			Stuffed Food Toy			Tug Play		
Behaviour Category	A	R	S	A	R	S	A	R	S	A	R	S	A	R	S	A	R	S	A	R	S
Compared to Bonding																					
Compared to Bubble machine																					
Compared to Conspecific play																					
Compared to Interactive toy																					
Compared to Playhouse																					
Compared to Stuffed food toy																					
Compared to Tug play																					

## Data Availability

The data presented in this study are available on request from the corresponding author subject to the agreement of the assistance dog organisation.

## Supplementary Materials

The following supporting information can be downloaded at: https://www.mdpi.com/article/10.3390/ani12020141/s1, Table S1: The behaviours coded within each behavioural category; Table S2: Mean behaviour change scores for relaxation, alert and stress behaviour for each EE activity; Table S3: Significance values for LSD post hoc pairwise analyses when comparing each EE activity to one another for alert (A), relaxation (R) and stress (S) behaviour categories.
