# Supplementary material for: Effects of Environmental Enrichment on Dog Behaviour: Pilot Study
Source: Animals (Basel). 2022 Jan 7;12(2):141. doi: 10.3390/ani12020141 (PMC8772568; doi:10.3390/ani12020141)
Supplement: Supplementary file 1 [file animals-12-00141-s001.zip › animals-1363146-SI.pdf]

animals

MDPI

Short Communication

Effects of environmental enrichment on dog behaviour: pilot study

Supplementary Tables.

Table S1. The behaviours coded within each behavioural category.

Behaviour category	Individual behaviours
Maintenance	Drinking, self-grooming, stretching, scratching, yawning, body shaking, panting.
Play	Tail wagging, tail chasing, dog bow, chewing toys.
Locomotion	Moving around the pen, reposition of body, rolling, crawling, head tossing, standing up.
Relaxation	Lying down, asleep, resting, curled up.
Alert	Sitting upright, looking, ears pricked up, tail up.
Stress	Whining, grumbling, whimpering, mumbling, barking, jumping upon pen bars, pawing, nosing or biting the pen, circling, pacing, excess panting, lip smacking, repeated yawning, tail between the legs.

Table S2. Mean behaviour change scores for relaxation, alert and stress behaviour for each EE activity.

EE activity	Maintenance	Play	Locomotion	Relaxation	Alert	Stress
Bonding	1.75	-0.05	-0.55	10.65	-7.40	-5.70
Bubble machine	4.10	0.95	-0.70	14.35	-10.40	-8.00
Conspecific play	20.25	0.05	-2.70	18.30	-16.00	-10.15
Interactive toy	2.50	1.40	-1.10	4.25	-4.40	-6.35
Playhouse	16.70	0.35	1.20	11.85	-8.30	-13.20
Stuffed food toy	1.75	-0.45	0.95	1.00	0.20	-1.55
Tug play	2.85	0.90	-1.60	11.05	-5.40	-12.30
Overall mean	7.13	0.45	-0.64	10.21	-7.39	-8.18

Table S3. Significance values for LSD post hoc pairwise analyses when comparing each EE activity to one another for alert (A), relaxation (R) and stress (S) behaviour categories. A green square indicates a significant increase for relaxation behaviours or a significant decrease for alert and stress behaviours. A red square indicates a significant decrease for relaxation behaviours or a significant increase for alert and stress behaviours

EE activity	Bonding			Bubble machine			Conspecific play			Interactive toy			Playhouse			Stuffed food toy			Tug play		
Behaviour category	A	R	S	A	R	S	A	R	S	A	R	S	A	R	S	A	R	S	A	R	S
Compared to Bonding				0.398	0.207	0.508	0.018	1.000	0.203	0.398	0.011	0.851	0.799	0.758	0.034	0.035	<0.001	0.234	0.572	1.000	0.061
Compared to Bubble machine	0.398	0.207	0.508				0.117	0.207	0.536	0.094	<0.001	0.634	0.553	0.338	0.138	0.004	<0.001	0.067	0.161	0.207	0.218
Compared to Conspecific play	0.018	1.000	0.203	0.117	0.207	0.536				0.002	0.011	0.276	0.033	0.758	0.381	<0.001	<0.001	0.016	0.004	1.000	0.536
Compared to Interactive toy	0.398	0.011	0.851	0.094	<0.001	0.634	0.002	0.011	0.276				0.273	0.005	0.052	0.197	0.214	0.170	0.777	0.011	0.090
Compared to Playhouse	0.799	0.758	0.034	0.553	0.338	0.138	0.033	0.758	0.381	0.273	0.005	0.052				0.019	<0.001	0.001	0.413	0.758	0.795
Compared to Stuffed food toy	0.035	<0.001	0.234	0.004	<0.001	0.067	<0.001	<0.001	0.016	0.197	0.214	0.170	0.019	<0.001	0.001				0.117	<0.001	0.003

Compared to Tug play	0.572	1.000	0.061	0.161	0.207	0.218	0.004	1.000	0.536	0.777	0.011	0.090	0.413	0.758	0.795	0.117	<0.001	0.003	
